# Selection signature reveals genes associated with susceptibility loci affecting respiratory disease due to pleiotropic and hitchhiking effect in Chinese indigenous pigs

**DOI:** 10.5713/ajas.18.0658

**Published:** 2019-02-07

**Authors:** Zhong Xu, Hao Sun, Zhe Zhang, Cheng-Yue Zhang, Qing-bo Zhao, Qian Xiao, Babatunde Shittu Olasege, Pei-Pei Ma, Xiang-Zhe Zhang, Qi-Shan Wang, Yu-Chun Pan

**Affiliations:** 1 Department of Animal Science, School of Agriculture and Biology, Shanghai Jiao Tong University, Shanghai 200240, China; 2 Shanghai Key Laboratory of Veterinary Bio-technology, Shanghai 200240, China

**Keywords:** Pigs, Selection Signature, Respiratory Disease, XP-EHH, F_ST_

## Abstract

**Objective:**

Porcine respiratory disease is one of the most important health problems causing significant economic losses. To understand the genetic basis for susceptibility to swine enzootic pneumonia (EP) in pigs, we detected 102,809 single nucleotide polymorphisms in a total of 249 individuals based on genome-wide sequencing data.

**Methods:**

Genome comparison of susceptibility to swine EP in three pig breeds (Jinhua, Erhualian, and Meishan) with two western lines that are considered more resistant (Duroc and Landrace) using cross-population extended haplotype homozygosity and F-statistic (F_ST_) statistical approaches identified 691 positively selected genes. Based on quantitative trait loci, gene ontology terms and literature search, we selected 14 candidate genes that have convincible biological functions associated with swine EP or human asthma.

**Results:**

Most of these genes were tested by several methods including transcription analysis and candidate genes association study. Among these genes: cytochrome P450 1A1 and catenin beta 1 (*CTNNB1*) are involved in fertility; transforming growth factor beta receptor 3 plays a role in meat quality traits; Wnt family member 2, *CTNNB1* and transcription factor 7 take part in adipogenesis and fat deposition simultaneously; plasminogen activator, urokinase receptor (completely linked to AXL receptor tyrosine kinase, r^2^ = 1) plays an essential role in the successful ovulation of matured oocytes in pigs; colipase like 2 (strongly linked to SAM pointed domain containing ETS transcription factor, r^2^ = 0.848) is involved in male fertility.

**Conclusion:**

These adverse genes susceptible to swine EP may be selected while selecting for economic traits (especially reproduction traits) due to pleiotropic and hitchhiking effect of linked genes. Our study provided a completely new point of view to understand the genetic basis for susceptibility or resistance to swine EP in pigs thereby, provides insight for designing sustainable breed selection programs. Finally, the candidate genes are crucial due to their potential roles in respiratory diseases in a large number of species, including human.

## INTRODUCTION

Porcine respiratory disease is one of the most important health problems associated with swine production [1]. It can be caused by various infectious pathogens, such as viruses, mycoplasma, bacteria and parasites. Swine enzootic pneumonia (EP) caused by *Mycoplasma hyopneumoniae* (*Mhp*) is a chronic and endemic respiratory disease, which has similar pathogenesis and clinical symptoms to human asthma [2]. *Mhp* infections are highly prevalent in almost all swine producing areas, causing significant economic losses due to decreased growth rate, poor feed efficiency and increased cost of healthcare. Both the control and eradication of EP are difficult because of its complicated mechanisms of infection and its co-infection with other respiratory pathogens such as *Mycoplasma hyorhinis*, *Pasteurella multocida*, porcine respiratory and reproductive syndrome virus (PRRSV), and porcine circovirus type 2. Many efforts including vaccination, medication and sanitation have been devoted to controlling its prevalence. However, the frequent application of antibiotics can increase the incidence of drug resistant bacteria and also raise the level of antibiotic residue in food.

Recently, the genetic improvement of disease resistance in animals has been attracting attention. Several studies have explored the genetic mechanisms of susceptibility to EP. Okamura et al [3] improved a Landrace breed by selecting for swine EP resistance and meat production over five generations. They identified a significant quantitative trait locus (QTL) for EP between microsatellite markers SW1650 and SW240 on S.scrofa chromosome 2. Huang et al [4] conducted a genome-wide association studies on 332 Chinese Erhualian pigs which were genotyped using Illumina Porcine 60K single nucleotide polymorphism (SNP) chips, and found C-X-C motif chemokine ligand 6 (*CXCL6*), *CXCL8*, KIT proto-oncogene, receptor tyrosine kinase, and C-terminal binding protein 2 as candidate genes that might play important roles in determining resistance or susceptibility to swine EP-like respiratory disease.

In the previous researches and practical production, we noticed that Chinese indigenous pig breeds such as Meishan pigs, Erhualian pigs, and Jinhua pigs are more sensitive to *Mhp* compared with commercial Western pig breeds in the same rearing environment [4,5]. By coincidence, these pig breeds show an excellent fertility and meat quality compared to European breeds and other Chinese breeds under the natural and artificial selective pressure. We proposed two hypotheses: One possibility is that the susceptible loci affecting swine EP are closely linked to the genes or regulatory elements involved in fertility and meat quality traits, and these genes or regulatory elements may have a hitch-hiking effect during the selection for fertility and meat quality traits; another possibility is that the pleiotropic genes or regulatory elements involved in fertility and meat quality traits might simultaneously play important roles in determining susceptibility to swine EP.

To test these hypotheses, we compared the genomes of three Chinese indigenous pig breeds which are very sensitive to swine EP with two commercial pig breeds using cross-population extended haplotype homozygosity (XP-EHH) and F-statistic (F_ST_) approaches [6,7] to detect genomic regions under positive selection that are related to susceptibility to swine EP. Recently, extensive researches have been carried to identify the positive selection of genes in relation to different specific traits using a distorted pattern of genetic variation between populations. For example, positive selection identified several candidate genes involved in Berkshire meat quality [8]. Taye et al [9] also discovered positively selected genes in African cattle responsible for thermotolerance, and Yuan et al [10] revealed several genes associated with tail type in Chinese indigenous sheep.

The aim of this study is to identify genes associated with susceptibility to respiratory disease like swine EP in Chinese indigenous populations using a genomic scan of selective sweep signatures, and to explore how it happened. Understanding the genetic mechanisms in pigs could help to design sustainable breed selection programs.

## MATERIALS AND METHODS

### Animals and genotyping

A total of 249 individuals were considered in this study. There were three pig breeds sensitive to swine EP consisting of 53 Jinhua pigs, 31 Erhualian pigs, and 80 Meishan pigs (50 Middle Meishan and 30 Small Meishan pigs), and two breeds relatively insensitive to swine EP including 48 Duroc pigs and 37 Landrace pigs. The sequencing data of Jinhua pigs were obtained from Li et al [11], Erhualian and Meishan pigs were retrieved from Wang et al [12], Duroc and Landrace pigs were acquired from Zhang et al [13]. All individuals were genotyped using the genotyping by genome reducing and sequencing (GGRS) protocol [14].

The Sscrofa10.2 pig reference genome was used for calling SNPs via SAMtools (version 0.1.19). Quality control of the fastq file was performed using NGS QC Toolkit v2.3, and the parameters were set according to the report from Chen et al [14]. Clean sequencing reads were subsequently mapped to the pig reference genome (Sscrofa10.2) using BWA v0.7.5 with default settings for single-end mapping. The SNP calling was performed using SAMtools software, and the missing genotypes were imputed using BEAGLE v4.1. Some filters were applied to SNPs as follows: i) minor allele frequency ≤0.05; ii) minimum number of individuals with genotyping ≤35%; and iii) SNPs on chromosome X. We filtered the SNPs on chromosome X in consideration of the extremely ancient interspecies introgression and low rate of recombination.

### Population structure analysis

To assess the relationships between the animals and breeds under investigation, several procedures were carried out. To generate a pruned subset of SNPs that are in approximate linkage equilibrium with each other in multidimensional scaling (MDS) analysis, the “--indep 50 5 2” command was used in PLINK v1.07 [15]. Principal component analysis (PCA) was performed using GCTA software (version 1.24), which represented the population structure based on genetic correlations between individuals. Pairwise identity-by-state distances between all individuals were calculated using PLINK.

### Genome scans for selection signatures

Before statistical analysis, Jinhua, Erhualian, and Meishan pigs were clustered into Group1 (observed group), whereas Duroc and Landrace pigs were clustered into Group2 (reference groups). To determine a genome wide pattern of positive selection between Group1 and Group2, F_ST_ values per-SNP were calculated based on the formulae proposed by Weir and Cockerham [7]. All F_ST_ values in this study are for a single locus. To determine if Group1 has undergone selection, we computed the XP-EHH values using haplotype information in the xpehh program [6]. The phased haplotype data was reconstructed using FASTPHASE (http://stephenslab.uchicago.edu/software.html). The XP-EHH values were estimated by calculating EHH and log-ratio iHH by comparing haplotypes between Group1 and Group2. EHH and REHH values were calculated using SWEEP v.1.1 (http://www.broadinstitute.org/mpg/sweep/) software.

### Gene annotation

We searched for positional candidate genes in a 20-kb region centering on the selected SNPs using the NCBI database and Ensembl pig genome databases (http://uswest.ensembl.org/Sus_scrofa/Info/Index). To further analyze the functions of identified genes, Kyoto encyclopedia of genes and genomes (KEGG) pathway and gene ontology (GO) enrichment analyses were performed using the Database for Annotation, Visualization and Integrated Discovery (DAVID). Due to the sparsity of the porcine gene database, we corresponded these genes with the human genome by aligning human ensemble ID. Only terms with a p-value (Benjamini correction) less than 0.05 were considered as significant and listed.

### Selection strategy for candidate genes involved in the susceptibility to swine enzootic pneumonia

To select genes that contribute to the susceptibility to swine EP from the total genes with strong signatures of selection, we used the following selection strategies: i) the pig QTL (SS_10.2) database was downloaded from the animal QTL database website (https://www.animalgenome.org/cgi-bin/QTLdb/SS/index). Because the number of QTLs of Mycoplasmal pneumonia susceptibility (MPS) trait is still relatively few, selection strategy; ii) used the keyword “Mycoplasma” find the MPS relevant traits, and then the genes overlapped with the QTLs were selected as groupA. Meanwhile, the genes matched to GO terms associated with lung, immune and inflammatory response were selected as groupB; iii) We merged these two groups and then the plausible genes were manually selected based on literature search, GeneCard and their biological function.

### Transcription analysis based on gene expression omnibus

To complement and verify the selected candidate genes, transcription analysis based on gene expression omnibus (GEO) was conducted. The only one published Expression profiling microarray (GSE49882, GPL17577) of the porcine alveolar macrophage infected by porcine *Mhp* was downloaded from public GEO database [16], and differentially expressed genes were identified by GEO2R using default parameters. A threshold was applied based on a fold change (FC) of 2.0 (p≤0.05) between infected (6h*Mhp*) and control (6hControl) cells.

### Candidate genes association study

In this analysis, all 171 Jinhua pigs were treated similarly. The individuals were genotyped using the same genotyping by GGRS protocol. The phenotypes were recorded based on Huang et al [4]. Briefly, one EP score was recorded for an individual which was found with a dry cough or obvious abdominal fluctuations and suffering from fast breathing and loss of appetite per day during 100 to 200 days of age. According to their EP scores, these pigs were grouped into controls (EP score = 0) and cases (EP score ≥1). Then we calculated the allele frequencies in the candidate genes between case and control groups and the p-values of SNPs were calculated by Fisher’s exact test.

### Calculation of r^2^

In order to search for potential genes linked to these candidate genes involved in the susceptibility to swine EP owing to the hitchhiking effect or lack of recombination, we calculated the linkage disequilibrium (LD) scores between SNPs located in these candidate genes and the other SNPs in selected regions. The r^2^ between two SNPs was then computed using PLINK v1.07 [15]. In this study, two genes were considered to be connected if the SNPs located in these two genes were physically close to each other and were subject to strong LD (usually measured by r^2^≥0.8).

## RESULTS AND DISCUSSION

### Single nucleotide polymorphism quality control

**Figure 1:**
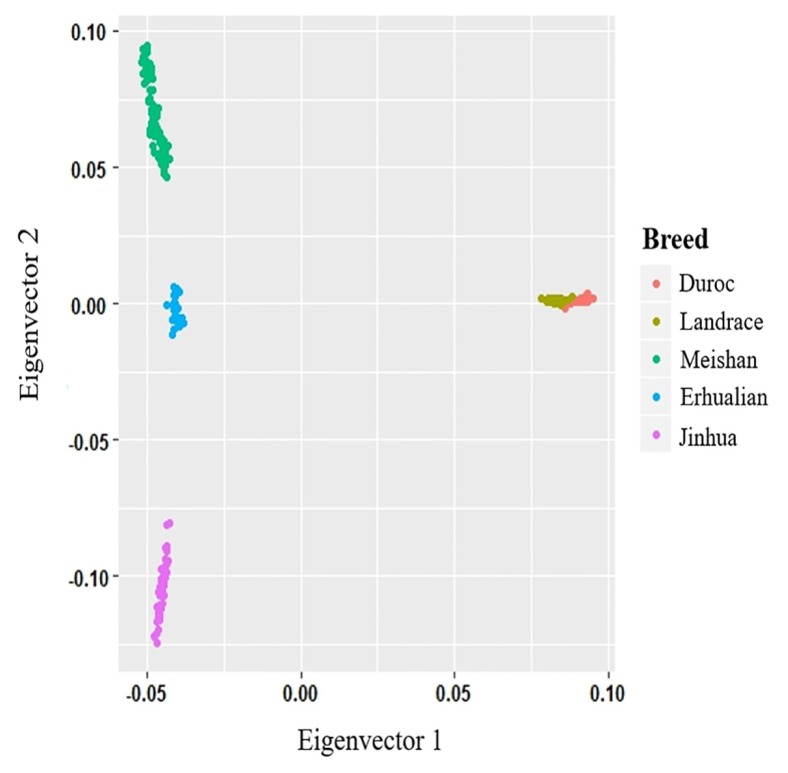
The distribution of the SNPs across the chromosomes. The x-axis denotes the chromosomal position (Mb), and the y-axis represents the chromosomes. The number of the SNPs present in each 1,000 kb genome block is expressed via colours. SNPs, single nucleotide polymorphisms.

After quality control, there were 249 subjects and 102,809 SNPs in the analyzed dataset. The variants were distributed on each chromosome in a relatively uniform fashion, with the exception of some isolated regions on some chromosomes (Figure 1). The SNPs positions within the chromosomes were based on the pig reference genome (SGSC Sscrofa10.2).

### Population structure

**Figure 2:**
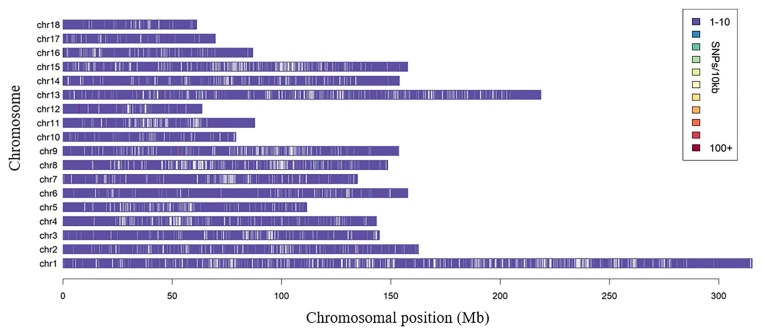
Population structure analysis of all individuals. Results of principal component analysis (PCA) of Duroc, Landrace, Jinhua, Erhualian, and Meishan breeds. Eigenvector1 (x-axis) versus Eigenvector2 (y-axis). The first two principal components, PC1 and PC2, account for 19.8% and 6.5% of the total variance, respectively.

To assess the relationships between the animals and breeds under investigation, we applied MDS to analyze 249 individuals using the pruned 35,104 SNPs with low LD extents. The PCA results showed that the first two principal components (PC1 and PC2) explained 19.8% and 6.5% of the variance respectively. The PC1 separated all individuals into two non-overlapping clusters (Figure 2). Our clustering results showed that the Jinhua, Erhualian, and Meishan pigs were grouped together as one group and that the Duroc, Landrace pigs belonged to another group.

### Positive selection signature of swine enzootic pneumonia sensitive populations

**Figure 3:**
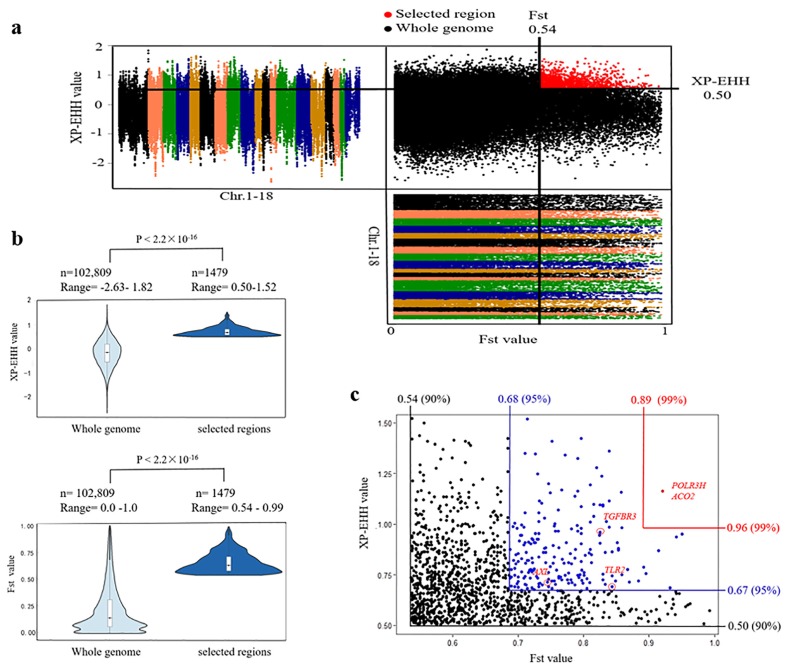
Genome-wide selective sweep analysis of group 1 (sensitive to swine enzootic pneumonia). (a) Distribution of XP-EHH and F_ST_. Data points located to the right of the vertical lines (10% right tails of the F_ST_ distribution, where F_ST_ is 0.54) and above the horizontal line (10% right tail of the XP-EHH distribution, where XP-EHH is 0.50) were identified as selected regions for group 1 pigs (red points); (b) Violin plot of XP-EHH and F_ST_ values for regions of group 1 pigs that have undergone positive selection versus the whole genome regions. The statistical significance was calculated by the Mann-Whitney U test. (c) 10%, 5%, and 1% significance level of XP-EHH and F_ST_. XP-EHH, cross-population extended haplotype homozygosity; F_ST_, F-statistic.

In this study, selection signature analysis was conducted using XP-EHH and F_ST_ approaches. We used an empirical procedure simultaneously with significantly high XP-EHH (10% right tail, where XP-EHH is 0.50) and F_ST_ values (10% right tail, where F_ST_ is 0.54) of the empirical distribution to clarify regions with strong selective sweep signals along the genome, which should harbor genes that underwent a selective sweep. Consequently, we identified a subset of 1,479 SNPs with strong selective sweep signals (Figure 3A), which exhibited significant differences (p<2.2×10^−16^, MannWhitney U test) in XP-EHH and F_ST_ values when compared to whole genomic background (Figure 3B). Finally, these SNPs represented 691 genes with strong signatures of selection (Supplementary Table S1), and only two genes, RNA polymerase III subunit H and aconitase 2 (*ACO2*), were embedded in the most significantly selected regions simultaneously with XP-EHH values (1% right tail) and F_ST_ values (1% right tail) (Figure 3C). *ACO2* was involved in tricarboxylic acid (TCA) cycle, and hypoxia, a fundamental characteristic of respiratory disease, can regulate the gene expression of mitochondrial aconitase [17].

Using the default settings within DAVID, GO terms, and KEGG pathways with p-value <0.05 were enriched and shown in Table 1. The terms overrepresented were related to cell adhesion, membrane part, membrane. In the KEGG analysis, 10 significant pathways were also identified: such as retinol metabolism, pentose and glucuronate interconversions, metabolism of xenobiotics by cytochrome P450, androgen and estrogen metabolism, starch and sucrose metabolism and porphyrin and chlorophyll metabolism.

### Positive selection signature related to susceptibility to swine enzootic pneumonia in Chinese indigenous pigs

While selective sweep signals are likely to be detected among various regions, in this paper, we focused on and discussed the genes and pathways that putatively contribute to the swine EP-susceptibility mechanisms of Chinese indigenous pigs. Among the 691 genes with strong signatures of selection, there are 50 genes located in seven QTLs of three traits (Mycoplasmal pneumonia susceptibility, *Mhp* antibody titer and Change in *Mhp* antibody titer) (Supplementary Table S2), and 33 genes matched the GO terms associated with lung, immune, inflammatory response and drug metabolism (Supplementary Table S3), and then we merged genes of these two groups together as preliminary candidate genes. After literature search based on GeneCard and their biological functions, a set of 14 genes that have convincible biological functions associated with swine EP or human asthma come into the priority candidate genes (Table 2 and Supplementary Table S4). Among these 14 genes, previous researches have reported cytochrome P450 1A1 (*CYP1A1*) and toll-like receptor 2 (*TLR2*) play an important role in the immune response to *Mhp* infection [18,19].

Innate immune response and inflammatory response play an important role in pathogenicity of swine EP. AXL receptor tyrosine kinase (*AXL*) regulates inhibition of toll-like receptors (TLRs)-mediated innate immune response and the DNA methylation of *AXL* at birth was associated with higher risk for asthma-related symptoms in early childhood [20]. C-C motif chemokine ligand 11 (*CCL11*), an inflammatory cytokine, was significantly related to increased risk of asthma in adults [21]. Interleukin 7 receptor (*IL7R*) may control the adaptive immune response to PRRSV vaccine in Pietrain pigs by transcriptome data. Kurz et al [22] identified five asthma susceptibility loci including IL7R in German and Hutterite populations.

The abnormality of Wnt/β-catenin signaling was believed to be associated with the development and pathogenesis of lung diseases. Wnt family member 2 (*WNT2*) and β-catenin 1 (*CTNNB1*) play a crucial role in the Wnt/β-catenin signaling pathway [23,24]. Transcription factor 7 (*TCF7*) is one of important transcription factors for T cell development and differentiation, tumorigenesis, or embryonic development [25]. It is suggested to be involved in immune responses to lung diseases such as pulmonary infection, asthma, acute lung injury, emphysema and lung cancer through several signal pathways, especially the canonical Wnt/β-catenin pathway [25].

The histochemistry and morphology of porcine airway cells would change in response to infection with swine EP. SAM pointed domain containing ETS transcription factor (*SPDEF*) selectively expressed in respiratory epithelia cells is an important transcriptional factor of airway goblet cell differentiation and pulmonary inflammation in response to aeroallergens, and it can regulate a transcriptional network mediating mucus secretion in chronic airway disease [26]. Matrix metallopeptidase 2 (*MMP2*), involved in the inflammatory response, can mediate IL-13-induced suppression of elastin expression in airway fibroblasts [27]. *CXCL2* and transforming growth factor beta receptor 3 (*TGFBR3*) play a role in the pathogenesis of airway remodeling in asthma [28].

Hypoxia is a fundamental component of respiratory dis ease, and the pressure of pulmonary and hematologic O_2_ and CO_2_ were decreased because of less oxygen consumption in swine with *Mhp* pneumonia [29]. Endothelial PAS domain protein 1 (*EPAS1*), also known as hypoxia inducible factor 2 subunit alpha, is a transcription factor that responds to hypoxia-responsive under high-altitude conditions in Tibetan pigs. Interestingly, a previous study reported that physiological hypoxia arises from lung alveoli suffering progressive airflow limitation which increases with pulmonary disease, chronic obstructive, severe early-onset (COPD) severity and *EPAS1* is a key regulator of COPD through responding to hypoxia induced by airflow limitation as proved by integrative analysis of DNA methylation and gene expression data [30]. *CYP1A2* is predominantly expressed in the liver which has been shown to modulate pulmonary oxygen toxicity. It plays a critical role in the susceptibility to hyperoxic lung injury by decreasing oxidative stress and lipid peroxidation in mice [31].

### Transcription analysis based on gene expression omnibus

**Figure 4:**
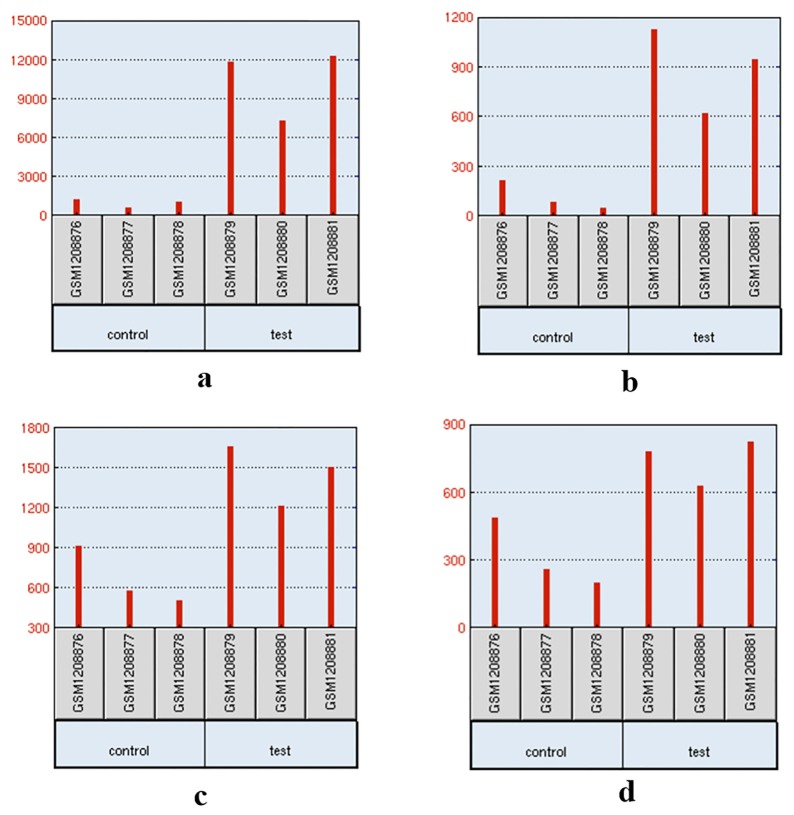
Expression of candidate genes in expression profiling array. (A) *CXCL2*, (B) *IL7R*, (C) *TLR2*, and (D) *EPAS1*. Transcription analysis of the porcine alveolar macrophage response to infection of *Mhp* between test (6h*Mhp*) and control (6hControl) cells. *CXCL2*, C-X-C motif chemokine ligand 2; *IL7R*, interleukin 7 receptor; *TLR2*, toll-like receptor 2; *EPAS1*, endothelial PAS domain protein 1; *Mhp*, *Mycoplasma hyopneumoniae.*

To complement and verify the candidate genes we selected, the allele frequencies of loci in these candidate genes between two groups are shown in Table 2. And the only one published microarray of the porcine alveolar macrophage infected by swine EP was collected from GEO database, and the candidate genes were analysed. The p-value and log_2_ (FC) were listed in Table 2. Among 14 candidate genes, only six genes were found in the expression profiling array (Platforms GPL17577). Four of the six genes, *EPAS1*, *CXCL2*, *TLR2*, and *IL7R*, were significantly up-regulated (Figure 4).

### Allele frequencies in candidate genes

After quantity control, we got 125,233 SNPs. Of the 171 Jinhua pigs, 93 pigs were grouped into controls (EP score = 0) and 78 pigs were grouped into cases (EP score ≥1). The allele frequencies in the 14 candidate genes were calculated between case and control groups. We found that the allele frequencies in six genes (*TCF7*, *TGFBR3*, *SPDEF*, *CCL11*, *IL7R*, and *WNT2*) were significantly different between those two groups (Supplementary Table S5). These results demonstrate the reliability of our results from another side.

### The pleiotropic genes may be related to swine enzootic pneumonia susceptibility

Among above genes, some have other biological function involved in fertility, meat quality traits, or adipogenesis and fat deposition simultaneously. *CYP1A1* can alter the activity of estrogen in the porcine ovary through estrogen receptor pathway [32]. *CYP1A1* plays an important role in the human placenta during pregnancy. *CTNNB1* as a molecular transcription factor is very important for the remodeling of uterine mucosa during development, the estrous cycle and early pregnancy in pigs [33]. *CTNNB1* contributes to intercellular adhesion in large antral follicles and might influence the normal follicle development and pig fertility [34]. *TGFBR3* plays a role in the muscular and adipose tissue development and it is a candidate gene involved in meat quality traits in pigs [8].

*WNT2*, *CTNNB1*, and *TCF7* are all involved in the Wnt/β-catenin signaling pathway, and this pathway plays a critical role in regulating porcine adipogenesis and fat deposition. Chinese indigenous breeds display a higher intramuscular fat content and more abdominal fat deposition than commercial Western pig breeds. And thus, these genes involved in fat deposition may be selected during the natural and artificial selection while they are simultaneously involved in susceptibility to EP disease in pigs. In a previous study, Huang et al also suggested that genes involved in fat deposition may play an important role in susceptibility or resistance to the EP disease in pigs [4]. Additionally, many epidemiologic studies have found that abdominal obesity increases the risk of developing asthma in humans [35].

### Linked genes with candidate genes may be related to swine enzootic pneumonia susceptibility

The LD scores between SNPs located in these candidate genes and the other SNPs in selected regions were calculated. Three genes subjected to strong LD (r^2^≥0.8) are listed in Table 3. Specifically, seven and two loci on chromosomes 6 were completely linked to *AXL* and *MMP2* respectively. In addition, two loci on chromosomes 7 were strongly linked to *SPDEF* (Table 3).

Plasminogen activator, urokinase receptor ( *PLAUR*) (completely linked to *AXL*) also known as CD87, is a part of the plasminogen activation system, which regulates the conversion of plasminogen to plasmin. *PLAUR* plays an essential role in the successful ovulation of matured oocytes together with follicle stimulating hormone in pigs [36]. Colipase like 2 (*CLPSL2*) (strongly linked to *SPDEF*, r^2^ = 0.848), specifically expressed in epididymis, was involved in the regulation of acrosomal integrity, spermatozoa motility, and male fertility. Knockdown of *CLPSL2* expression in mouse epididymis results in a decreased number of sperm with intact acrosome, attenuated sperm motility, and reduced the fertility [37].

We noticed that these Chinese indigenous pig breeds show an excellent fertility and meat quality, higher intramuscular fat content and more abdominal fat deposition compared to European breeds under the natural and artificial selective pressure. These adverse genes of susceptibility to swine EP may be selected simultaneously while selecting economic traits due to pleiotropic effects and hitchhiking effect of linked genes. These results support our hypothesis to a great extent and provide a completely new point of view to explain why Chinese indigenous pig breeds are more sensitive to swine EP.

In conclusion, this study revealed several candidate genes that are involved in the susceptibility to swine EP including *TCF7*, *EPAS1*, *TGFBR3*, *MMP2*, *AXL*, *SPDEF*, *CYP1A1*, *CYP1A2*, *CXCL2*, *TLR2*, *CCL11*, *CTNNB1*, *IL7R*, and *WNT2* from positive selection signature. Most of these genes are involved in inflammatory response, hypoxia-responsive or Wnt/β-catenin signaling pathway. The susceptibility loci affecting swine EP have increased during the selection for fertility and meat quality traits because of pleiotropic and hitch-hiking effects. These findings will help in increasing our understanding of the genetic basis for susceptibility or resistance to EP and other respiratory diseases in pigs thereby, provide insight for designing sustainable breed selection programs (such as marker assisted selection). Moreover, we provided a completely new point of view to explain why Chinese indigenous pig breeds are more sensitive to swine EP. In addition, exploring the molecular mechanisms of the susceptibility to swine EP may be crucial for its potential role in human asthma and other respiratory diseases.

## Supplementary Data











## Figures and Tables

**Table 1:** GO terms and KEGG pathways enriched with candidate genes

Category	Term	Gene number	p-value (Benjamini)
GO:0000267	Cell fraction	65	0.0022
GO:0044425	Membrane part	274	0.0127
GO:0016020	Membrane	296	0.0194
KEGG:hsa00830	Retinol metabolism	13	0.0001
KEGG:hsa00040	Pentose and glucuronate interconversions	7	0.0023
KEGG:hsa00053	Ascorbate and aldarate metabolism	7	0.0023
KEGG:hsa00150	Androgen and estrogen metabolism	8	0.0155
KEGG:hsa00980	Metabolism of xenobiotics by cytochrome P450	10	0.0190
KEGG:hsa00983	Drug metabolism	8	0.0282
KEGG:hsa00500	Starch and sucrose metabolism	8	0.0284
KEGG:hsa00860	Porphyrin and chlorophyll metabolism	7	0.0304
KEGG:hsa00140	Steroid hormone biosynthesis	8	0.0329
KEGG:hsa00982	Drug metabolism	9	0.0419

GO, gene ontology; KEGG, Kyoto encyclopedia of genes and genomes.

**Table 2:** Transcription analysis based on gene expression omnibus

Candidate genes	Chr	Physical position	Group11)		Group21)		Microarray	
		
			AF1	AF2	AF1	AF2	p-value	Log_2_(FC)
*TCF7*	2	142047175	0	1	0.488	0.512	NF	NF
*EPAS1*	3	100164175	0.024	0.976	0.694	0.306	3.38E-03	1.32
*TGFBR3*	4	136718160	0.018	0.982	0.806	0.194	NF	NF
*MMP2*	6	27552563	0.003	0.997	0.5	0.5	NF	NF
*AXL*	6	44979578	0.324	0.676	0.997	0.003	7.32E-01	0.08
*SPDEF*	7	35242328	0.128	0.872	0.8	0.2	NF	NF
*CYP1A1*	7	63474840	0.003	0.997	0.741	0.259	NF	NF
*CYP1A2*	7	63474840	0.003	0.997	0.741	0.259	NF	NF
*CXCL2*	8	74284832	0	1	0.529	0.471	3.10E-06	3.32
*TLR2*	8	79818077	0.04	0.96	0.871	0.129	2.53E-03	1.15
*CCL11*	12	42446570	0.476	0.524	0	1	NF	NF
*CTNNB1*	13	27684730	0	1	0.512	0.488	3.97E-01	0.24
*IL7R*	16	22326963	0.318	0.682	1	0	1.36E-04	3.08
*WNT2*	18	30813337	0.11	0.89	0.771	0.229	NF	NF

AF, allele frequency; FC, fold change; NF, not found; *TCF7*, transcription factor 7; *EPAS1*, endothelial PAS domain protein 1; *TGFBR3*, transforming growth factor beta receptor 3; *MMP2*, matrix metallopeptidase 2; *AXL*, AXL receptor tyrosine kinase; *SPDEF*, SAM pointed domain containing ETS transcription factor; *CYP1A1*, cytochrome P450 1A1; *CXCL2*, C-X-C motif chemokine ligand 2; *TLR2*, toll-like receptor 2; *CCL11*, C-C motif chemokine ligand 11; *CTNNB1*, catenin beta 1; *IL7R*, interleukin 7 receptor; *WNT2*, Wnt family member 2.

1) Group1, Jinhua, Erhualian and Meishan pigs; Group2, Duroc and Landrace pigs.

**Table 3:** Strong LD between SNPs located in candidate genes and the other SNPs in selected regions

Chr	Physical position	Genes	Chr	Physical position	LD (r^2^)	Associated genes
6	27552563	*MMP2*	6	27451454	1	*LPCAT2, SLC6A2*
			6	27451485	1	*LPCAT2, SLC6A2*
6	44979578	*AXL*	6	45100366	1	*CCDC97, HNRNPUL1*
			6	45487407	1	*MEGF8*
			6	45601909	1	*GSK3A, CYP2B6*
			6	46185803	1	*IRGQ, XRCC1, ZNF576*
			6	46185804	1	*IRGQ, XRCC1, ZNF576*
			6	46185819	1	*IRGQ, XRCC1, ZNF576*
			6	46331807	1	*PLAUR, CADM4*
7	35242328	*SPDEF*	7	35722329	0.881	*ANKS1A*
			7	36516210	0.848	*CLPSL2, ARMC12*

LD, linkage disequilibrium; SNPs, single nucleotide polymorphisms; *MMP2*, matrix metallopeptidase 2; *LPCAT2*, lysophosphatidylcholine acyltransferase 2; *SLC6A2*, solute carrier family 6 member 2; *AXL*, AXL receptor tyrosine kinase; *CCDC97*, coiled-coil domain containing 97; *HNRNPUL1*, heterogeneous nuclear ribonucleoprotein U like 1; *MEGF8*, multiple EGF like domains 8; *GSK3A*, glycogen synthase kinase 3 alpha; *CYP2B6*, cytochrome P450 family 2 subfamily B member 6; *IRGQ*, immunity related GTPase Q; *XRCC1*, X-ray repair cross complementing 1; *ZNF576*, zinc finger protein 576; *PLAUR*, plasminogen activator, urokinase receptor; *CADM4*, cell adhesion molecule 4; *SPDEF*, SAM pointed domain containing ETS transcription factor; *ANKS1A*, ankyrin repeat and sterile alpha motif domain containing 1A; *CLPSL2*, colipase like 2; *ARMC12*, armadillo repeat containing 12.
