# Supplementary material for: Selection signature reveals genes associated with susceptibility loci affecting respiratory disease due to pleiotropic and hitchhiking effect in Chinese indigenous pigs
Source: Asian-Australas J Anim Sci. 2019 Feb 7;33(2):187–96. doi: 10.5713/ajas.18.0658 (PMC6946968; doi:10.5713/ajas.18.0658)
Supplement: Supplementary file 1 [file ajas-18-0658-suppl1.pdf]

Selection signature analysis reveals genes associated with susceptibility loci affecting respiratory disease due to pleiotropic and hitchhiking effect in Chinese indigenous pigs

Zhong Xu[1], Hao Sun[1], Zhe Zhang[1], Cheng-Yue Zhang[1], Qing-bo Zhao[1], Qian Xiao[1], Babatunde Shittu Olasege[1], Pei-Pei Ma[1], Xiang-Zhe Zhang[1], Qi-Shan Wang[1*], Yu-Chun Pan[1, 2*]

[1] Department of Animal Science, School of Agriculture and Biology, Shanghai Jiao Tong University, Shanghai 200240, PR China

[2] Shanghai Key Laboratory of Veterinary Bio-technology, Shanghai 200240, PR China

Running title: The research of swine EP in pigs

*Corresponding author: Yuchun Pan; Qishan Wang.

E-mail: panyuchun1963@aliyun.com; wangqishan@sjtu.edu.cn.

Department of Animal Science, School of Agriculture and Biology, Shanghai Jiao Tong University, No.800 Dongchuan Road, Minhang District,Shanghai 200240, People's Republic of China

Phone: +86-21-34205836;

Fax: +86-21-34206394

Table S1. List of candidate genes resulted from genome-wide positive selection scan (overlapped in XP-EHH and F_{ST}).

A2M	ADIPOR1	ARHGAP18	C1QTNF5	CCDC71	COL27A1	CYP1A1	DSN1
ABAT	ADM	ARHGEF3	C20orf194	CCDC97	CORIN	CYP1A2	DYRK1B
ABCA8	ADO	ARL15	C20orf202	CCK	COX8A	CYP2B6	EBF4
ABCA9	AGL	ARMC7	C21orf62	CCL11	CPNE8	CYP46A1	ECE1
ABCC8	AGXT	ARPC1A	C2orf54	CCL8	CPT1C	CYP4A11	EDIL3
ABHD11	AK5	ARSI	C3orf67	CD109	CPXM1	CYP4A22	EEA1
ABLIM2	AK7	ASRGL1	C6	CD19	CRB1	DAP3	EFCAB8
ABR	AKT1S1	ATG7	C6orf47	CD84	CRELD2	DAPK2	EFTUD2
ABTB1	ALDH16A1	ATL3	CACHD1	CD8B	CRISPLD2	DCDC2B	EGFLAM
ABTB2	ALG6	ATM	CACNA1I	CDH15	CSDC2	DCUN1D3	EGR2
ACACB	AMPD1	ATOH1	CADM4	CDK17	CSNK2B	DDX24	EHHADH
ACO2	AMZ1	ATP2A1	CADPS	CHPF	CST11	DDX4	EIF3I
ACPT	ANKFY1	ATP2A3	CADPS2	CHRM1	CST8	DENND1B	EIF4G3
ACSL1	ANKRD33B	ATP8A2	CALML4	CHST10	CST9L	DGCR8	ELOVL7
ACSM2A	ANKS1A	ATRN	CALML6	CIC	CSTL1	DHX32	ENO4
ACSM2B	ANO1	AXL	CALU	CLCN1	CTNNA2	DIO2	ENPP6
ACSM5	ANTXR2	B3GAT2	CAPNS2	CLDN4	CTNNB1	DLK1	EPAS1
ACTB	ANXA9	B4GALNT2	CAPSL	CLEC16A	CTSB	DNAJB4	ERF
ACTL7A	AOAH	BBS2	CARHSP1	CLN6	CTU1	DNAJC30	ESAM
ACTL7B	AP2A1	BCL2L12	CASP2	CLSTN3	CWF19L2	DNER	ESPL1
ACVR2A	AP2A2	BSDC1	CC2D2A	CMIP	CXCL1	DNHD1	EXTL1
ADAM12	APOM	BTBD11	CCDC103	CNIH3	CXCL2	DOCK2	FADD
ADAM33	AQP7	BTD	CCDC116	CNOT6L	CXCL3	DPEP2	FAHD2A
ADAMTS12	ARAP2	C16orf72	CCDC158	CNTN3	CYB5D2	DPEP3	FAHD2B
ADAMTS13	ARFGEF1	C1orf115	CCDC30	COL16A1	CYB5R1	DRD5	FAM13A
FAM160A2	GATA2	GXYLT2	IGLV3-10	ITPR1	LDLRAD3	MEX3A	NCKAP1L

FAM188B	*GDAP1L1*	*HABP2*	*IGLV3-12*	*JAG2*	*LENG8*	*MFAP3L*	*NDUFA10*
FAM200A	*GFAP*	*HACL1*	*IGLV3-16*	*KCNN3*	*LILRA4*	*MFRP*	*NFX1*
FAM46C	*GFRA4*	*HAS1*	*IGLV3-19*	*KCTD10*	*LILRA5*	*MFSD5*	*NISCH*
FAM63A	*GGT1*	*HDHD2*	*IGLV3-21*	*KDM5B*	*LILRA6*	*MGLL*	*NKAIN2*
FAM71F1	*GGT2*	*HEATR5A*	*IGLV3-22*	*KIAA0556*	*LILRB3*	*MKI67*	*NKX3-2*
FANK1	*GGTLC1*	*HECTD1*	*IGLV3-25*	*KIAA0922*	*LILRB4*	*MLXIPL*	*NOSIP*
FBL	*GGTLC2*	*HEPACAM*	*IGLV3-27*	*KIF17*	*LILRB5*	*MMAA*	*NPFFR1*
FBRSL1	*GGTLC3*	*HERC3*	*IGLV3-32*	*KIF18B*	*LMNA*	*MMEL1*	*NPHP1*
FBXL18	*GIPC2*	*HHIPL1*	*IGLV3-9*	*KIF1A*	*LNX1*	*MMP2*	*NR1H2*
FCGBP	*GMPPA*	*HHLA1*	*IGSF21*	*KLHL29*	*LPCAT2*	*MPP7*	*NSUN5*
FCGRT	*GNA12*	*HIRA*	*IL12RB2*	*KLK4*	*LRIG1*	*MRC2*	*NT5C*
FDFT1	*GNAS*	*HN1*	*IL17REL*	*KRCC1*	*LRRC4C*	*MRPL42*	*NT5DC2*
FEZF1	*GNB1L*	*HNRNPUL1*	*IL21R*	*KRT14*	*LRRK1*	*MSTO1*	*NUP214*
FKBP5	*GON4L*	*HOXC10*	*IL4I1*	*KRT19*	*LTBP1*	*MT1X*	*NUP54*
FKBP6	*GPAT2*	*HOXC11*	*IL6R*	*KRT31*	*LTF*	*MT3*	*NXN*
FLT3LG	*GPATCH8*	*HOXC6*	*IL7R*	*KRT33A*	*LY6G5B*	*MUC6*	*OLFML1*
FOXI1	*GPR18*	*HOXC8*	*ILK*	*KRT33B*	*LY6G5C*	*MYBL2*	*OLFML2B*
FOXK1	*GPR182*	*HP1BP3*	*IQCC*	*KRT34*	*MACROD1*	*MYBPC2*	*OR1A1*
FOXN4	*GREB1*	*HRH1*	*IRF3*	*KSR2*	*MAL*	*MYO10*	*OR1A2*
FRMD4B	*GRIA4*	*HTR2A*	*IRGQ*	*L3MBTL2*	*MAN2A1*	*MYO7A*	*OR1L4*
FSHR	*GRIP1*	*IARS*	*IRX6*	*LAMA2*	*MAP3K14*	*NAAA*	*OR1L6*
FUZ	*GRIP2*	*ICAM2*	*ITGA5*	*LAMB2*	*MAVS*	*NADSYN1*	*OR52N1*
FXYD1	*GRM5*	*IDO1*	*ITGB2*	*LARS2*	*MBOAT1*	*NAGK*	*OR52N5*
FYB	*GSG1L*	*IFT52*	*ITIH4*	*LAT*	*MED25*	*NAT10*	*OR5P2*
GABRD	*GSK3A*	*IGLV3-1*	*ITPA*	*LDB2*	*MEGF8*	*NCF2*	*OR9A2*
OSBPL10	*PMEPA1*	*PTOV1*	*RNY4P19*	*SDK2*	*SLC4A11*	*SNORD42B*	*TAF10*
OTOP1	*PMM1*	*PTPRE*	*ROBO4*	*SEC24B*	*SLC4A7*	*SNORD4A*	*TAF4*
OTUB1	*PNKP*	*RAB25*	*RPH3A*	*SEMA5A*	*SLC5A1*	*SNORD88A*	*TAF6L*

OTUB2	*PODXL2*	*RAB34*	*RPL23A*	*SETDB1*	*SLC6A2*	*SNORD88B*	*TAS2R41*
OVCH2	*POFUT2*	*RABEP1*	*RPS11*	*SFMBT1*	*SLC9A9*	*SNORD88C*	*TBC1D17*
PACSIN1	*POLD1*	*RABEP2*	*RPS3A*	*SH2B1*	*SLIT1*	*SNPH*	*TBC1D4*
PAFAH1B3	*POLR2G*	*RAI14*	*RRP8*	*SHANK1*	*SLIT3*	*SNX18*	*TBL2*
PAFAH2	*POLR3H*	*RAMP1*	*RTL1*	*SHANK2*	*SMUG1*	*SNX27*	*TBX3*
PANK4	*POM121*	*RAP1GAP2*	*RTN3*	*SIGLEC14*	*SMYD1*	*SNX9*	*TCF7*
PARD3	*POM121C*	*RASAL2*	*RUFY2*	*SIGLEC5*	*SNORA19*	*SPATA17*	*TCOF1*
PCDH15	*PPA1*	*RASGEF1B*	*RUFY3*	*SIPA1L1*	*SNORA25*	*SPDEF*	*TDRD3*
PDGFRB	*PPEF2*	*RBAK*	*RYR2*	*SIPA1L2*	*SNORA6*	*SPRYD3*	*TEX261*
PDS5A	*PPP1R14C*	*RBP2*	*SBF2*	*SLAMF1*	*SNORD113-1*	*ST3GAL1*	*TGFBR3*
PGA3	*PRKAR2A*	*RBP5*	*SBK1*	*SLAMF6*	*SNORD113-7*	*ST6GALNAC4*	*THADA*
PGA4	*PRKCA*	*RCN3*	*SCAP*	*SLC10A2*	*SNORD113-9*	*ST6GALNAC6*	*THBD*
PGA5	*PRKCDBP*	*RDH16*	*SCARA5*	*SLC12A4*	*SNORD22*	*STAB1*	*THNSL2*
PIAS1	*PRKCE*	*REEP3*	*SCARB2*	*SLC16A5*	*SNORD26*	*STARD4*	*THRB*
PIH1D1	*PRLR*	*REXO4*	*SCARNA18*	*SLC18A2*	*SNORD27*	*STK32A*	*TIMM22*
PIK3R5	*PRMT1*	*RFC1*	*SCARNA20*	*SLC22A10*	*SNORD28*	*STOX1*	*TLR2*
PITRM1	*PROCA1*	*RFTN1*	*SCGB2A1*	*SLC25A20*	*SNORD30*	*STX1A*	*TMEM110*
PLA2G16	*PRPF38A*	*RHOH*	*SCGB2A2*	*SLC25A38*	*SNORD32A*	*STX5*	*TMEM138*
PLAUR	*PRPSAP2*	*RIMS4*	*SCN4A*	*SLC26A2*	*SNORD32B*	*SUPT6H*	*TMEM139*
PLCH2	*PRR19*	*RNF150*	*SCN5A*	*SLC39A11*	*SNORD33*	*SUSD4*	*TMEM145*
PLCXD3	*PRRG2*	*RNF217*	*SCNN1B*	*SLC39A8*	*SNORD34*	*SYT12*	*TMEM154*
PLD4	*PSMD5*	*RNF26*	*SDAD1*	*SLC3A2*	*SNORD35A*	*SYT9*	*TMEM156*
PLEKHM1	*PSMD9*	*RNPEPL1*	*SDF2L1*	*SLC4A1*	*SNORD35B*	*TAC3*	*TMEM167A*
TMEM200B	*TRIM73*	*UBE2F*	*UGT2B7*	*VPS16*	*WDR64*	*ZBTB3*	*ZNF576*
TMEM216	*TRIM74*	*UBE2L3*	*UHMK1*	*VPS37D*	*WDR66*	*ZBTB39*	*ZNF655*
TMEM5	*TRPC7*	*UBE3B*	*ULK1*	*VSIG2*	*WDR74*	*ZCCHC11*	*ZNF789*
TMEM52	*TTC27*	*UBR4*	*USP10*	*WBSCR22*	*WNT2*	*ZNF175*	*ZP4*
TOX2	*TTC9C*	*UGT2B10*	*USP19*	*WBSCR27*	*XPO6*	*ZNF226*	*ZYG11B*

TPCN1	*TTLL8*	*UGT2B11*	*USP2*	*WBSCR28*	*XRCC1*	*ZNF268*
TPP1	*TULP4*	*UGT2B15*	*USP4*	*WDHD1*	*XRCC4*	*ZNF385A*
TRAK1	*TXN2*	*UGT2B17*	*VANGL2*	*WDR11*	*YDJC*	*ZNF385D*
TRAPPC9	*TYR*	*UGT2B28*	*VAX2*	*WDR19*	*YY1AP1*	*ZNF395*
TRIM50	*UBAC2*	*UGT2B4*	*VGLL4*	*WDR36*	*ZAP70*	*ZNF526*

Table S2. List of candidate genes in QTLs associated with swine EP susceptibility. MPS: Mycoplasmal pneumonia susceptibility; MHT: Mycoplasma hyopneumoniae antibody titer; MHTC: Change in Mycoplasma hyopneumoniae antibody titer.

Trait Name	QTL_ID	Chr	Start	End	genes
MPS	21314	12	35033156	47927603	*CCL11, CCL8, PROCA1, RAB34, RPL23A, SNORD42B, SNORD4A, SUPT6H*
MHT	12317	2	38367026	151463344	*ABCC8, ADM, EDIL3, MAN2A1, SBF2, SCARNA18, SLC25A48, TCF7, TRPC7, WDR36, XRCC4*
	12329	18	24222671	46327930	*AOAH, CADPS2, FEZF1, WNT2*
	12330	18	603581	7783092	*CASP2, CLCN1, OR9A2, TAS2R41, TMEM139*
MHTC	12316	2	133375944	151463344	*SLC25A48, TCF7, TRPC7*
	12328	16	342954	80539062	*ADAMTS12, ARL15, C6, CAPSL, DDX4, DOCK2, EGFLAM, ELOVL7, FOXI1, FYB, MYO10,PRLR, RAI14, RNU6-768P, SEMA5A, SLIT3, SNX18*
	12331	18	603581	7783092	*CASP2, CLCN1, OR9A2, TAS2R41, TMEM139*
