# Supplementary material for: Selection signature reveals genes associated with susceptibility loci affecting respiratory disease due to pleiotropic and hitchhiking effect in Chinese indigenous pigs
Source: Asian-Australas J Anim Sci. 2019 Feb 7;33(2):187–96. doi: 10.5713/ajas.18.0658 (PMC6946968; doi:10.5713/ajas.18.0658)
Supplement: Supplementary file 2 [file ajas-18-0658-suppl2.pdf]

TPCN1	*TTLL8*	*UGT2B11*	*USP2*	*WBSCR28*	*XRCC1*	*ZNF268*
TPP1	*TULP4*	*UGT2B15*	*USP4*	*WDHD1*	*XRCC4*	*ZNF385A*
TRAK1	*TXN2*	*UGT2B17*	*VANGL2*	*WDR11*	*YDJC*	*ZNF385D*
TRAPPC9	*TYR*	*UGT2B28*	*VAX2*	*WDR19*	*YY1AP1*	*ZNF395*
TRIM50	*UBAC2*	*UGT2B4*	*VGLL4*	*WDR36*	*ZAP70*	*ZNF526*

Table S2. List of candidate genes in QTLs associated with swine EP susceptibility. MPS: Mycoplasmal pneumonia susceptibility; MHT: Mycoplasma hyopneumoniae antibody titer; MHTC: Change in Mycoplasma hyopneumoniae antibody titer.

Trait Name	QTL_ID	Chr	Start	End	genes
MPS	21314	12	35033156	47927603	*CCL11, CCL8, PROCA1, RAB34, RPL23A, SNORD42B, SNORD4A, SUPT6H*
MHT	12317	2	38367026	151463344	*ABCC8, ADM, EDIL3, MAN2A1, SBF2, SCARNA18, SLC25A48, TCF7, TRPC7, WDR36, XRCC4*
	12329	18	24222671	46327930	*AOAH, CADPS2, FEZF1, WNT2*
	12330	18	603581	7783092	*CASP2, CLCN1, OR9A2, TAS2R41, TMEM139*
MHTC	12316	2	133375944	151463344	*SLC25A48, TCF7, TRPC7*
	12328	16	342954	80539062	*ADAMTS12, ARL15, C6, CAPSL, DDX4, DOCK2, EGFLAM, ELOVL7, FOXI1, FYB, MYO10,PRLR, RAI14, RNU6-768P, SEMA5A, SLIT3, SNX18*
	12331	18	603581	7783092	*CASP2, CLCN1, OR9A2, TAS2R41, TMEM139*
