# Supplementary material for: Selection signature reveals genes associated with susceptibility loci affecting respiratory disease due to pleiotropic and hitchhiking effect in Chinese indigenous pigs
Source: Asian-Australas J Anim Sci. 2019 Feb 7;33(2):187–96. doi: 10.5713/ajas.18.0658 (PMC6946968; doi:10.5713/ajas.18.0658)
Supplement: Supplementary file 3 [file ajas-18-0658-suppl3.pdf]

**Table S3. List of candidate genes matched the GO terms.**

| Chr | Gene Start | Gene End | Gene Name | GO Term Name |
|---|---|---|---|---|
| 2 | 3185891 | 3194845 | *FADD* | immune system process |
| 2 | 3185891 | 3194845 | *FADD* | innate immune response |
| 2 | 3185891 | 3194845 | *FADD* | positive regulation of adaptive immune response |
| 2 | 114675917 | 114853524 | *MAN2A1* | lung alveolus development |
| 3 | 18629655 | 18643387 | *LAT* | inflammatory response |
| 3 | 18629655 | 18643387 | *LAT* | immune response |
| 3 | 56515143 | 56541806 | *ZAP70* | innate immune response |
| 3 | 94167752 | 94253662 | *EPAS1* | lung development |
| 3 | 94351279 | 94864954 | *PRKCE* | macrophage activation involved in immune response |
| 4 | 90051204 | 90080747 | *VANGL2* | orthogonal dichotomous subdivision of terminal units involved in lung branching morphogenesis |
| 4 | 90051204 | 90080747 | *VANGL2* | planar dichotomous subdivision of terminal units involved in lung branching morphogenesis |
| 4 | 90051204 | 90080747 | *VANGL2* | lateral sprouting involved in lung morphogenesis |
| 4 | 125109966 | 125310783 | *TGFBR3* | immune response |
| 6 | 30058595 | 30087031 | *MMP2* | positive regulation of innate immune response |
| 6 | 49110789 | 49178413 | *CYP2B6* | drug metabolic process |
| 6 | 49245802 | 49277917 | *AXL* | inflammatory response |
| 6 | 49638991 | 49660975 | *CIC* | lung alveolus development |
| 7 | 30512173 | 30524271 | *SPDEF* | lung goblet cell differentiation |
| 7 | 58779475 | 58786117 | *CYP1A2* | lung development |
| 7 | 58779475 | 58786117 | *CYP1A2* | drug metabolic process |
| 7 | 58802880 | 58809775 | *CYP1A1* | drug metabolic process |
| 8 | 70054756 | 70163415 | *CXCL2* | inflammatory response |
| 8 | 70054756 | 70163415 | *CXCL2* | immune response |
| 8 | 75416028 | 75428330 | *TLR2* | positive regulation of inflammatory response |
| 8 | 75416028 | 75428330 | *TLR2* | inflammatory response |

| | | | | |
|---|---|---|---|---|
| 8 | 75416028 | 75428330 | *TLR2* | cytokine secretion involved in immune response |
| 8 | 75416028 | 75428330 | *TLR2* | immune system process |
| 8 | 75416028 | 75428330 | *TLR2* | innate immune response |
| 8 | 112670533 | 112762870 | *SEC24B* | lung morphogenesis |
| 8 | 112670533 | 112762870 | *SEC24B* | lung lobe morphogenesis |
| 9 | 36620587 | 36759552 | *ATM* | immune system process |
| 10 | 20392326 | 20707474 | *DENND1B* | regulation of immune response |
| 12 | 18142322 | 18184103 | *MAP3K14* | immune response |
| 12 | 40738450 | 40782893 | *CCL11* | inflammatory response |
| 12 | 40738450 | 40782893 | *CCL11* | immune response |
| 12 | 46709880 | 46895818 | *ABR* | negative regulation of inflammatory response |
| 13 | 25159603 | 25208313 | *CTNNB1* | lung development |
| 13 | 25159603 | 25208313 | *CTNNB1* | epithelial tube branching involved in lung morphogenesis |
| 13 | 25159603 | 25208313 | *CTNNB1* | lung cell differentiation |
| 13 | 25159603 | 25208313 | *CTNNB1* | lung-associated mesenchyme development |
| 13 | 25159603 | 25208313 | *CTNNB1* | lung induction |
| 13 | 25159603 | 25208313 | *CTNNB1* | mesenchymal cell proliferation involved in lung development |
| 13 | 25159603 | 25208313 | *CTNNB1* | positive regulation of branching involved in lung morphogenesis |
| 13 | 29411457 | 29445953 | *LTF* | innate immune response in mucosa |
| 13 | 29411457 | 29445953 | *LTF* | antimicrobial humoral immune response mediated by antimicrobial peptide |
| 13 | 67209260 | 67411314 | *HRH1* | inflammatory response |
| 14 | 53577131 | 53584546 | *ZP4* | positive regulation of humoral immune response |
| 16 | 19547007 | 19920513 | *ADAMTS12* | regulation of inflammatory response |
| 16 | 26023986 | 26083742 | *C6* | immune response |
| 16 | 53843251 | 54249181 | *DOCK2* | myeloid dendritic cell activation involved in immune response |
| 17 | 9245135 | 9261748 | *IDO1* | cytokine production involved in inflammatory response |
| 17 | 9245135 | 9261748 | *IDO1* | positive regulation of chronic inflammatory response |
| 17 | 9245135 | 9261748 | *IDO1* | inflammatory response |

| | | | | |
|---|---|---|---|---|
| 17 | 9245135 | 9261748 | *IDO1* | positive regulation of type 2 immune response |
| 18 | 28946497 | 29028107 | *WNT2* | positive regulation of epithelial cell proliferation involved in lung morphogenesis |
| 18 | 28946497 | 29028107 | *WNT2* | lung induction |
| 18 | 37377924 | 37599931 | *AOAH* | negative regulation of inflammatory response |

**Table S4. Information of genes which are previously reported as with swine EP or human asthma related genes. Descriptions of the gene functions are based on literature search and GeneCard.**

| Candidate genes | Chr | Physical position | XP-EHH score | Fst value | Function | Reference |
|---|---|---|---|---|---|---|
| *TCF7* | 2 | 142047175 | 0.75 | 0.58 | Involved in the Wnt signaling pathway and immune response. | (Zhu *et al*. 2015) |
| *EPAS1* | 3 | 100164175 | 0.57 | 0.72 | Responds to hypoxia-responsive. | (Ai *et al*. 2014) |
| *TGFBR3* | 4 | 136718160 | 0.96 | 0.83 | Airway remodeling. | (Kim *et al*. 2010) |
| *MMP2* | 6 | 27552563 | 1.02 | 0.58 | Plays crucial role in the inflammatory response. | (Huang *et al*. 2009) |
| *AXL* | 6 | 44979578 | 0.71 | 0.75 | Mediates innate immune response. | (Sun *et al*. 2010) |
| *SPDEF* | 7 | 35242328 | 0.95 | 0.64 | Mediates mucus secretion in airway. | (Rajavelu *et al*. 2015) |
| *CYP1A1* | 7 | 63474840 | 0.54 | 0.80 | Responds to inflammatory response caused by M.hyopneumoniae. | (Fang *et al*. 2016) |
| *CYP1A2* | 7 | 63474840 | 0.54 | 0.80 | Susceptibility to hyperoxic lung injury. | (Wang *et al*. 2015) |
| *CXCL2* | 8 | 74284832 | 0.75 | 0.62 | Inflammation response and the pathogenesis of airway remodeling. | (Al-Alwan *et al*. 2013) |
| *TLR2* | 8 | 79818077 | 0.69 | 0.84 | Responds to inflammatory response caused by M.hyopneumoniae. | (Fang *et al*. 2013) |
| *CCL11* | 12 | 42446570 | 0.66 | 0.57 | Significantly related to increased risk of asthma. | (Zhou *et al*. 2017) |
| *CTNNB1* | 13 | 27684730 | 0.73 | 0.60 | Plays crucial role in the Wnt/β-catenin signaling pathway. | (Smolich *et al*. 1993) |
| *IL7R* | 16 | 22326963 | 0.58 | 0.76 | Responds to adaptive immune response. | (Kurz *et al*. 2006) |
| *WNT2* | 18 | 30813337 | 0.54 | 0.63 | Involved in the Wnt signaling pathway and immune response. | (Smolich *et al*. 1993) |
