# Supplementary material for: Selection signature reveals genes associated with susceptibility loci affecting respiratory disease due to pleiotropic and hitchhiking effect in Chinese indigenous pigs
Source: Asian-Australas J Anim Sci. 2019 Feb 7;33(2):187–96. doi: 10.5713/ajas.18.0658 (PMC6946968; doi:10.5713/ajas.18.0658)
Supplement: Supplementary file 4 [file ajas-18-0658-suppl4.pdf]

17	9245135	9261748	*IDO1*	positive regulation of type 2 immune response
18	28946497	29028107	*WNT2*	positive regulation of epithelial cell proliferation involved in lung morphogenesis
18	28946497	29028107	*WNT2*	lung induction
18	37377924	37599931	*AOAH*	negative regulation of inflammatory response

Table S4. Information of genes which are previously reported as with swine EP or human asthma related genes. Descriptions of the gene functions are based on literature search and GeneCard.

Candidate genes	Chr	Physical position	XP-EHH score	Fst value	Function	Reference
TCF7	2	142047175	0.75	0.58	Involved in the Wnt signaling pathway and immune response.	(Zhu *et al*. 2015)
EPAS1	3	100164175	0.57	0.72	Responds to hypoxia-responsive.	(Ai *et al*. 2014)
TGFBR3	4	136718160	0.96	0.83	Airway remodeling.	(Kim *et al*. 2010)
MMP2	6	27552563	1.02	0.58	Plays crucial role in the inflammatory response.	(Huang *et al*. 2009)
AXL	6	44979578	0.71	0.75	Mediates innate immune response.	(Sun *et al*. 2010)
SPDEF	7	35242328	0.95	0.64	Mediates mucus secretion in airway.	(Rajavelu *et al*. 2015)
CYP1A1	7	63474840	0.54	0.80	Responds to inflammatory response caused by M.hyopneumoniae.	(Fang *et al*. 2016)
CYP1A2	7	63474840	0.54	0.80	Susceptibility to hyperoxic lung injury.	(Wang *et al*. 2015)
CXCL2	8	74284832	0.75	0.62	Inflammation response and the pathogenesis of airway remodeling.	(Al-Alwan *et al*. 2013)
TLR2	8	79818077	0.69	0.84	Responds to inflammatory response caused by M.hyopneumoniae.	(Fang *et al*. 2013)
CCL11	12	42446570	0.66	0.57	Significantly related to increased risk of asthma.	(Zhou *et al*. 2017)
CTNNB1	13	27684730	0.73	0.60	Plays crucial role in the Wnt/β-catenin signaling pathway.	(Smolich *et al*. 1993)
IL7R	16	22326963	0.58	0.76	Responds to adaptive immune response.	(Kurz *et al*. 2006)
WNT2	18	30813337	0.54	0.63	Involved in the Wnt signaling pathway and immune response.	(Smolich *et al*. 1993)
