# Supplementary material for: Selection signature reveals genes associated with susceptibility loci affecting respiratory disease due to pleiotropic and hitchhiking effect in Chinese indigenous pigs
Source: Asian-Australas J Anim Sci. 2019 Feb 7;33(2):187–96. doi: 10.5713/ajas.18.0658 (PMC6946968; doi:10.5713/ajas.18.0658)
Supplement: Supplementary file 5 [file ajas-18-0658-suppl5.pdf]

Table S5 Allele frequencies in candidate genes

Candidate genes	Chr	Physical position	Control		Case		P-value
			Allele 1	Allele 2	Allele 1	Allele 2	
TCF7	2	141987756	20	166	4	152	0.00279
	2	141987781	167	19	150	6	0.03524
TGFBR3	4	136805034	94	92	49	107	0.00042
	4	136821106	104	82	109	47	0.00990
	4	136899780	96	90	49	107	0.00019
	4	136901227	102	84	53	103	0.00013
SPDEF	7	35209896	175	11	154	2	0.04293
	7	35227275	175	11	154	2	0.04293
	7	35246393	9	177	0	156	0.00455
CCL11	12	42413773	126	60	81	75	0.00381
	12	42413807	93	93	57	99	0.01598
	12	42413821	93	93	57	99	0.01598
IL7R	16	22330173	110	76	124	32	0.00007
	16	22331566	77	109	84	72	0.02286
	16	22331570	79	107	84	72	0.03933
WNT2	18	30772891	123	63	75	81	0.00095
	18	30772914	124	62	76	80	0.00094
	18	30772943	124	62	77	79	0.00139
	18	30777492	184	2	137	19	0.00001

Ai H, Yang B, Li J, Xie X, Chen H, Ren J. 2014. Population history and genomic signatures for high-altitude adaptation in Tibetan pigs. *BMC genomics* **15**: 834.

Al-Alwan LA, Chang Y, Mogas A, Halayko AJ, Baglole CJ, Martin JG, Rousseau S, Eidelman DH, Hamid Q. 2013. Differential roles of CXCL2 and CXCL3 and their receptors in regulating normal and asthmatic airway smooth muscle cell migration. *Journal of immunology* **191**: 2731-2741.

Fang X, Liu X, Meng C, Fu Y, Wang X, Li B, Tu F, Zhao F, Ren S. 2013. Breed-linked polymorphisms of porcine toll-like receptor 2 (TLR2) and TLR4 and the primary investigation on their relationship with prevention against Mycoplasma pneumoniae and bacterial LPS challenge. *Immunogenetics* **65**: 829-834.

Fang X, Zhao W, Xu J, Tu F, Wang X, Li B, Fu Y, Ren S. 2016. CYP1A1 mediates the suppression of major inflammatory cytokines in pulmonary alveolar macrophage (PAM) cell lines caused by Mycoplasma hyponeumoniae. *Developmental and comparative immunology* **65**: 132-138.

Huang H, Zhao W, Tang Z, Yang S, Wu Z, Zhao S, Cui W, Mu Y, Chu M, Li K. 2009. Characterization of porcine MMP-2 and its association with immune traits. *Gene* **435**: 63-71.

Kim HK, Jang TW, Jung MH, Park HW, Lee JE, Shin ES, Cho SH, Min KU, Kim YY. 2010. Association between genetic variations of the transforming growth factor ss receptor type III and asthma in a Korean population. *Experimental & molecular medicine* **42**: 420-427.

Kurz T, Hoffjan S, Hayes MG, Schneider D, Nicolae R, Heinzmann A, Jerkic SP, Parry R, Cox NJ, Deichmann KA et al. 2006. Fine mapping and positional candidate studies on chromosome 5p13 identify multiple asthma susceptibility loci. *The Journal of allergy and clinical immunology* **118**: 396-402.

Rajavelu P, Chen G, Xu Y, Kitzmiller JA, Korfhagen TR, Whitsett JA. 2015. Airway epithelial SPDEF integrates goblet cell differentiation and pulmonary Th2 inflammation. *The Journal of clinical investigation* **125**: 2021-2031.

Smolich BD, McMahon JA, McMahon AP, Papkoff J. 1993. Wnt family proteins are secreted and associated with the cell surface. *Molecular biology of the cell* **4**: 1267-1275.

Sun B, Qi N, Shang T, Wu H, Deng T, Han D. 2010. Sertoli cell-initiated testicular innate immune response through toll-like receptor-3 activation is negatively regulated by Tyro3, Axl, and mer receptors. *Endocrinology* **151**: 2886-2897.

Wang L, Lingappan K, Jiang W, Couroucli XI, Welty SE, Shivanna B, Barrios R, Wang G, Firoze Khan M, Gonzalez FJ et al. 2015. Disruption of cytochrome P4501A2 in mice leads to increased susceptibility to hyperoxic lung injury. *Free radical biology & medicine* **82**: 147-159.

Zhou T, Huang X, Zhou Y, Ma J, Zhou M, Liu Y, Xiao L, Yuan J, Xie J, Chen W. 2017. Associations between Th17-related inflammatory cytokines and asthma in adults: A Case-Control Study. *Scientific reports* **7**: 15502.

Zhu Y, Wang W, Wang X. 2015. Roles of transcriptional factor 7 in production of inflammatory factors for lung diseases. *Journal of translational medicine* **13**: 273.
